# Supplementary material for: rNMPID: a database for riboNucleoside MonoPhosphates in DNA
Source: Bioinform Adv. 2024 May 8;4(1):vbae063. doi: 10.1093/bioadv/vbae063 (PMC11088741; doi:10.1093/bioadv/vbae063)
Supplement: vbae063_Supplementary_Data [file vbae063_supplementary_data.zip › Supplementary_fig_3_26_24 FS.pptx]

## Slide 1
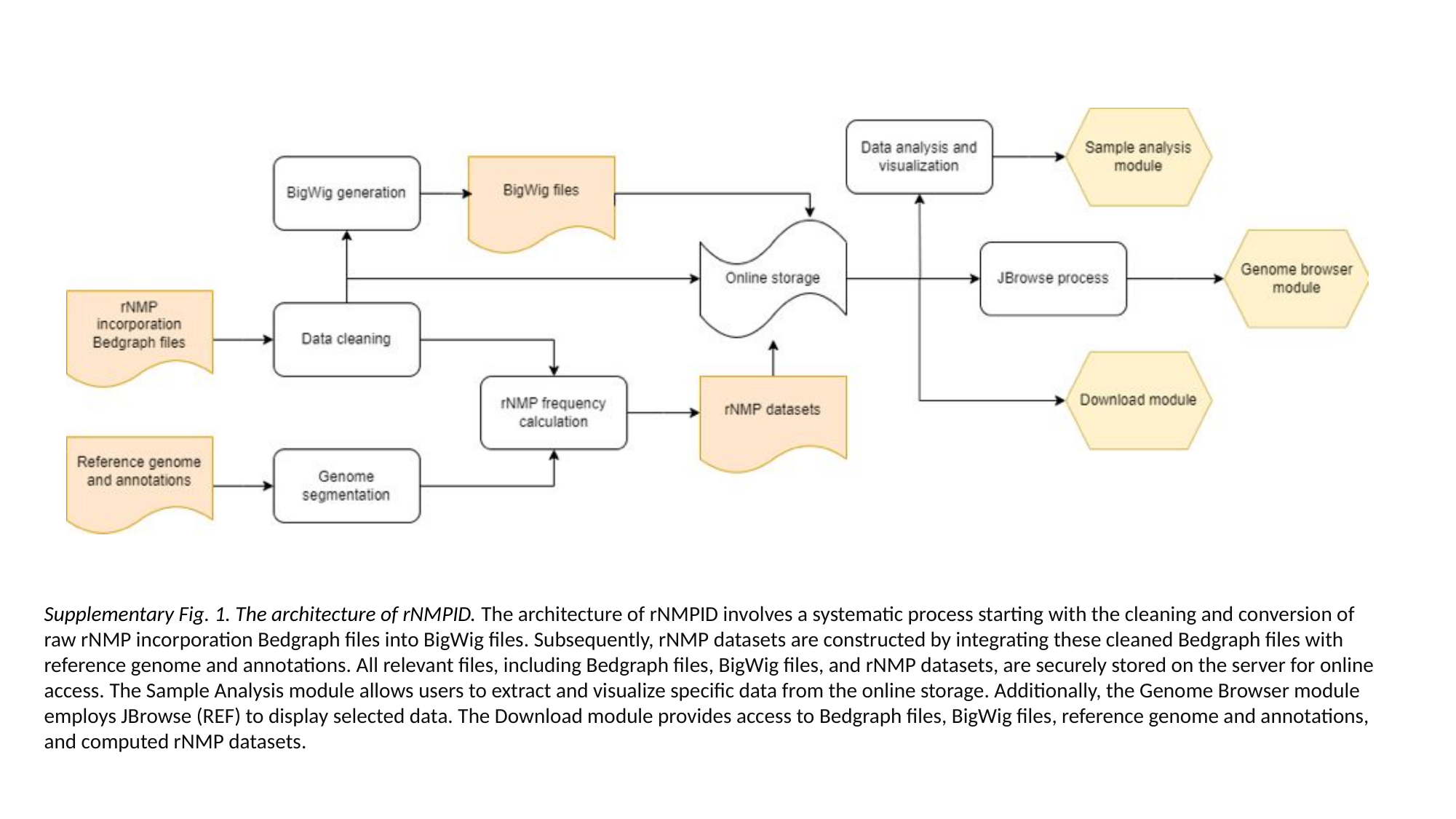

Supplementary Fig. 1. The architecture of rNMPID. The architecture of rNMPID involves a systematic process starting with the cleaning and conversion of raw rNMP incorporation Bedgraph files into BigWig files. Subsequently, rNMP datasets are constructed by integrating these cleaned Bedgraph files with reference genome and annotations. All relevant files, including Bedgraph files, BigWig files, and rNMP datasets, are securely stored on the server for online access. The Sample Analysis module allows users to extract and visualize specific data from the online storage. Additionally, the Genome Browser module employs JBrowse (REF) to display selected data. The Download module provides access to Bedgraph files, BigWig files, reference genome and annotations, and computed rNMP datasets.

## Slide 2
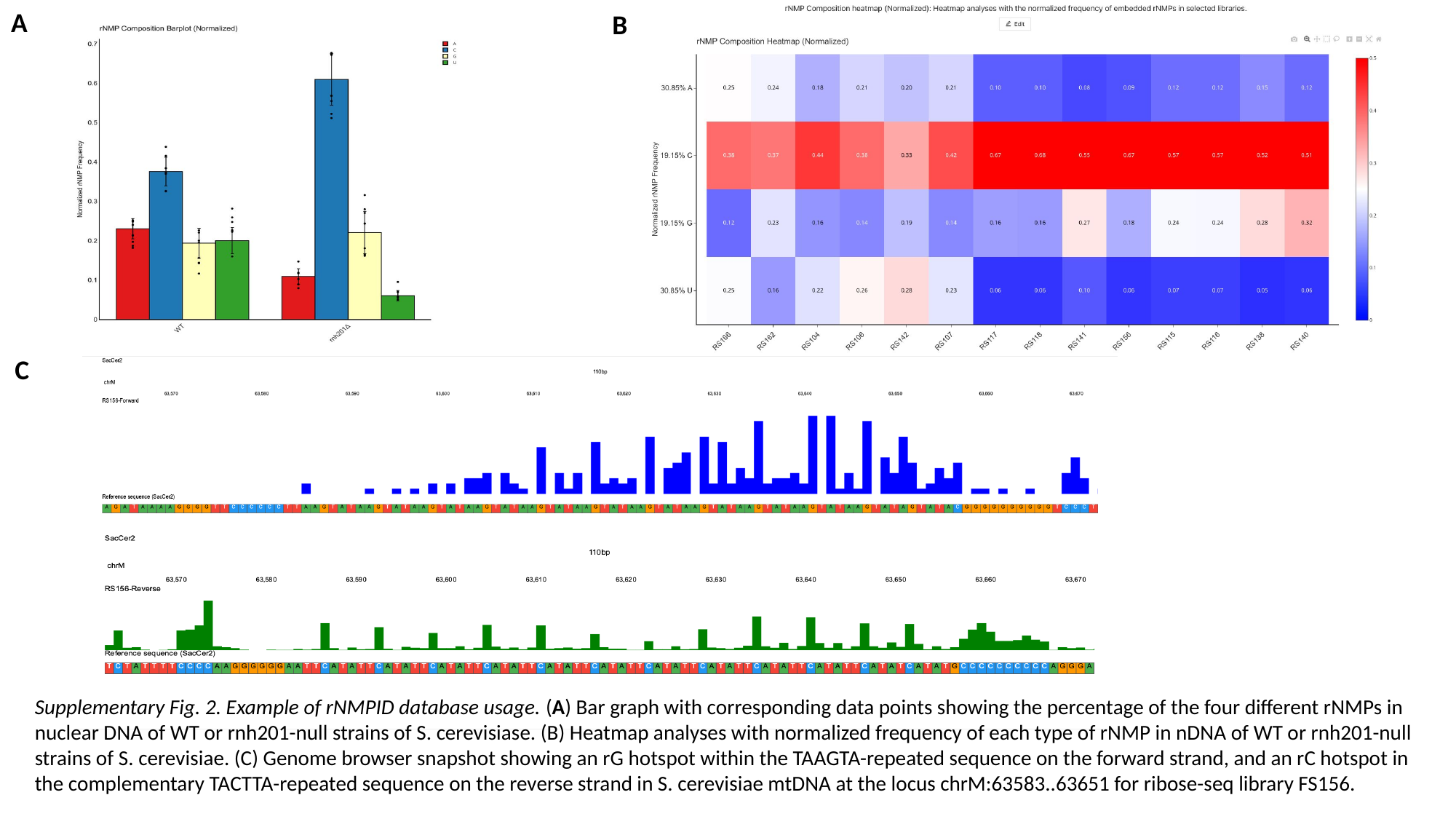

A
B
C
Supplementary Fig. 2. Example of rNMPID database usage. (A) Bar graph with corresponding data points showing the percentage of the four different rNMPs in nuclear DNA of WT or rnh201-null strains of S. cerevisiase. (B) Heatmap analyses with normalized frequency of each type of rNMP in nDNA of WT or rnh201-null strains of S. cerevisiae. (C) Genome browser snapshot showing an rG hotspot within the TAAGTA-repeated sequence on the forward strand, and an rC hotspot in the complementary TACTTA-repeated sequence on the reverse strand in S. cerevisiae mtDNA at the locus chrM:63583..63651 for ribose-seq library FS156.
