# Supplementary material for: rNMPID: a database for riboNucleoside MonoPhosphates in DNA
Source: Bioinform Adv. 2024 May 8;4(1):vbae063. doi: 10.1093/bioadv/vbae063 (PMC11088741; doi:10.1093/bioadv/vbae063)
Supplement: vbae063_Supplementary_Data [file vbae063_supplementary_data.zip › supplementary_table_20240311 FS 3_26_24.docx]

**Supplementary Table S1 All rNMP libraries in the rNMPID database**

| Technique | Species | Number of libraries | Reference genome version | Genome coverage | Accession Number | Source |
| --- | --- | --- | --- | --- | --- | --- |
| ribose-seq | *Homo sapiens* | 32 | Hg38 | Mitochondria | PRJNA941970 | Xu et al., 2024 |
|  | *Saccharomyces cerevisiae* | 39 | SacCer2 | Whole genome | PRJNA613920;  GSE240399 | Balachander et al., 2020  Kundnani et al. |
|  | *Saccharomyces paradoxus* | 4 | ASM207905v1 | Whole genome | PRJNA613920 | Balachander et al., 2020 |
|  | *Schizosaccharomyces pombe* | 5 | Pombase | Whole genome | PRJNA613920 | Balachander et al., 2020 |
|  | *Chlamydomonas reinhardtii* | 3 | Creinhardtii_281_v5 | Whole genome | PRJNA657931 | El-Sayed et al., 2021 |
| emRiboSeq | *Mus musculus* | 14 | mm9 with additional circularisation junction of the mitochondrial genome | Whole genome | GSE103429 | Moss et al., 2017 |
|  | *Saccharomyces cerevisiae* | 20 | sacCer3 (SGD version 64) | Whole genome | GSE64521 | Reijns et al., 2015 |
| Alk-HydEn-seq | *Saccharomyces cerevisiae* | 29 | L03 reference v2 | Whole genome | GSE62181 | Clausen et al., 2015 |
|  | *Saccharomyces cerevisiae* | 82 | UCSC (sacCer3) | Whole genome | GSE100352GSE110241 | Wanrooij et al., 2017  Kreisel et al., 2019 |
|  | *Saccharomyces cerevisiae* | 28 | W303 background RNR1 assembly | Whole genome | GSE101698 | Garbacz et al., 2018 |
|  | *Escherichia coli* | 12 | DH10B | Whole genome | GSE141315 | Łazowski et al., 2023 |
| RHII-HydEn-seq | *Saccharomyces cerevisiae* | 34 | L03 | Whole genome | GSE125855 | Zhou et al., 2019 |
|  | *Schizosaccharomyces pombe* | 24 | S. pombe assembly 2.23 | Whole genome | GSE125855 | Zhou et al., 2019 |
